# Prognostic factors of patients with recurrent uterine malignancies undergoing secondary cytoreductive surgery

**DOI:** 10.1186/s12905-023-02708-2

**Published:** 2024-01-02

**Authors:** Chenyan Fang, Yingli Zhang, Ping Zhang, Tao Zhu

**Affiliations:** Department of Gynecological Oncology, Zhejiang Cancer Hospital, Hangzhou Institute of Medicine (HIM), Chinese Academy of Sciences, 1 Banshan East Road, Hangzhou, 310022 Zhejiang Province China

**Keywords:** Recurrent uterine malignancy, Secondary cytoreductive Surgery, Prognosis

## Abstract

**Background:**

Several studies have demonstrated that secondary cytoreductive surgery (SCS) for patients with recurrent uterine malignancies may improve the survival. However, the selection criteria for SCS remain to be defined. This study aimed to assess the outcome of SCS and to explore factors that may influence the prognosis.

**Methods:**

Data of patients with recurrent uterine malignancies who received SCS in our hospital between January 2005 and January 2015 were retrospectively analyzed. Patients were assigned into endometrial carcinoma (EC) group and uterine sarcoma (US) group.

**Results:**

84 cases in total were involved in the study, including 47 cases with recurrent EC and 37 cases with recurrent US. The 5-year survival of cases with recurrent EC and recurrent US was 59.6% and 33.3%, respectively. Recurrent EC cases with a lower tumor grade (G1/G1-G2/G2), size of the largest tumor ≤ 6 cm, single recurrent tumor, a history of adjuvant therapy, as well as recurrent US cases with younger age, a longer disease-free interval (DFI) before SCS (≥ 12 months), no peritoneal dissemination, and a history of complete cytoreduction were associated with a longer survival. The number of recurrent tumors was found as an independent prognostic factor of SCS.

**Conclusion:**

Recurrent EC cases with a lower tumor grade, smaller tumor size, single tumor, a history of adjuvant therapy, as well as recurrent US cases with younger age, a longer DFI before SCS, no peritoneal dissemination, and a history of complete cytoreduction were more likely to benefit from SCS.

## Background

Uterine malignancies include endometrial carcinoma (EC) and uterine sarcoma (US). EC is the second most common gynecological malignancy in China, and the most common cancer in developed countries [1, 2]. Although EC cases are mainly diagnosed in early-stage and have a good prognosis, approximately 11–13% of EC cases develop recurrence with a mortality rate of about 25% [3, 4]. Recurrent EC cases may undergo radiotherapy, chemotherapy, surgery, molecular targeted therapy or hormone therapy. Optimal management of recurrent EC has not been well defined, while several retrospective studies have demonstrated that secondary cytoreductive surgery (SCS) for recurrent EC could improve the survival in a select patient population [5–8].

In addition, US accounts for about 1% of all gynecological malignancies and 3 ~ 7% of all uterine cancers [9]. The recurrence rate of US has been reported to be as high as 50–70% [10]. After recurrence, radiotherapy, chemotherapy, surgery or hormone therapy can also be selected, and molecular targeted therapies for US have been mainly presented in clinical trials. Due to the scarcity and the histopathological heterogeneity, it is difficult to determine the optimal management for US [11]. Some retrospective studies have also demonstrated that SCS for recurrent US could improve the survival in a select patient population [12–16].

Previous studies have shown that cases with endometrioid histology, isolated site of recurrence, Eastern Cooperative Oncology Group (ECOG) performance status score of 0, complete resection, time to the first recurrence > 12 months, age < 70 years old upon initial diagnosis, and high histology grade were associated with a longer survival after SCS [17–19]. Among them, complete resection was the most influential factor, and young age, solitary recurrent tumor, tumor size < 6 cm, no peritoneal dissemination, and ECOG performance status score of 0 were predictors of optimal surgical resection [6, 17].

Recurrent EC and US cases with specific characteristic may benefit from SCS. Now we performed a retrospective analysis of this issue again in our center to access the outcome of SCS in these patients and explore factors that may influence the prognosis. In order to provide some help in the selection of patients for SCS.

## Methods

### Patients

Data of all cases with recurrent uterine malignancies who received SCS in Zhejiang Cancer Hospital (Hangzhou, China) between January 2005 and January 2015 were retrospectively analyzed, and 84 cases in total were enrolled in the present study.

The study was approved by the Medical Ethics Committee of Zhejiang Cancer Hospital. Written informed consent was waived since the study was retrospective. Data were harvested from medical records or by out-patient/ telephone interviews, including ECOG performance status, age, clinicopathological features, characteristics of recurrence, SCS data, follow-up data, etc.

### Follow-up

Cases were followed up at the first 1–2 months after the therapy, then followed by every 3 months for 2 years, every 6 months for 2–5 years, and once a year thereafter. Gynecological examination, abdominal ultrasonography, magnetic resonance imaging (MRI), computed tomography (CT) scan, positron emission tomography (PET), or biopsy was performed in each follow-up. And the recurrent disease was determined by gynecological imaging examination or biopsy. Overall survival (OS), which was defined as the duration from SCS to death or the last follow-up, and progression-free survival (PFS), which was defined as the duration from SCS to recurrence or the last follow-up, were used to evaluate survival of the cases.

### Statistical analysis

SPSS 17.0 statistical software (IBM, Armonk, NY, USA) was used for statistical analysis. Categorical data were analyzed using Fisher’s exact test or Chi-square test. The effects of different covariates on OS and PFS were analyzed using univariate and multivariate Cox regression models, which were expressed as hazard ratio (HR). The Kaplan-Meier method was used to plot survival curves, and the difference in survival was assessed by log-rank test. Univariate and multivariate logistic regression analyses of clinicopathological factors related to SCS outcomes were conducted. A two-sided P < 0.05 was considered statistically significant.

## Results

### Patients’ characteristics

A total of 84 cases with recurrent uterine malignancies were involved in this study, including 47 cases with recurrent EC and 37 cases with recurrent US.

The features of cases in the two groups are shown in Table 1. More than 85% of cases in the two groups had an ECOG performance status score of 0. More than 70% of cases in the two groups were in Federation of Gynecology and Obstetrics (FIGO) stage I. The most common histology was endometrioid carcinoma (80.9%) in the EC group and uterine leiomyosarcoma (51.4%) in the US group. More than 80% of cases in the two groups received the first treatment in other hospitals. The median disease-free interval (DFI) after primary treatment in the EC and US groups was 14 and 11 months, respectively.

**Table 1:** Clinicopathologic characteristics

	Endometrial Carcinoma(N%)	Uterine Sarcoma(N%)
Total	47	37
Age at recurrence,years (mean, range)	55 ± 9.441 (29–76)	50.92 ± 10.652 (34–74)
ECOG performance status before SCS		
0	41 (87.2)	32 (86.5)
1	5 (10.6)	4 (10.8)
2	1 (2.1)	1 (2.7)
FIGO stage at initial diagnosis		
I	33 (70.2)	28 (75.7)
II	2 (4.3)	4 (10.8)
III	9 (19.1)	4 (10.8)
IV	3 (6.4)	1 (2.7)
Histology		
Endometrioid	38 (80.9)	NA
G1	9 (23.7)	NA
G1-G2	5 (13.2)	NA
G2	5 (13.2)	NA
G2-G3	7 (18.4)	NA
G3	12 (31.6)	NA
Serous	3 (6.4)	NA
Clear cell	1 (2.1)	NA
Carcinosarcoma	5 (10.6)	NA
Uterine leiomyosarcoma	NA	19 (51.4)
Low-grade endometrial stromal sarcoma	NA	11 (29.7)
High-grade endometrial stromal sarcoma	NA	3 (8.1)
Other	NA	4 (10.8)
Primary treatment		
Surgery	100 (100)	36 (97.3)
Chemotherapy	0 (0)	1 (2.7)
Primary treatment in our hospital		
Yes	9 (19.1)	4 (10.8)
No	38 (80.9)	33 (89.2)
Adjuvant treatment after first surgery		
None	29 (61.7)	28 (75.7)
Chemotherapy only	7 (14.9)	7 (18.9)
Radiotherapy only	6 (12.8)	0 (0)
Combined chemoradiotherapy	2 (4.3)	0 (0)
Hormonal therapy	3 (6.4)	1 (2.7)
Other	0 (0)	1 (2.7)
DFI, months (median, range)	14 (1–96)	11 (2-109)

ECOG, Eastern Cooperative Oncology Group; SCS, Secondary cytoreductive surgery; FIGO, International Federation of Gynecology and Obstetrics; DFI, Disease-free interval

Characteristics of recurrence and SCS in the two groups are shown in Table 2. In cases with recurrent US, the incidence rate of multiple recurrent tumors (64.9% vs. 48.9%, P = 0.186) was slightly higher than that in cases with recurrent EC. In addition, compared with cases with recurrent EC, those with recurrent US had significantly more intraoperative blood loss and hemoglobin drop, longer hospital stays after SCS, and higher proportions of the largest tumor size > 6 cm (48.6% vs. 12.8%). Nearly half of the US cases (45.9%) had peritoneal dissemination. After SCS, the rate of complete cytoreduction in cases with recurrent EC was significantly higher than that in cases with recurrent US (80.9% vs. 54.1%). Additionally, the recurrence (89.2% vs. 55.3%) and death (75.7% vs. 44.7%) were more frequent among cases with recurrent US.

**Table 2:** Characteristics of recurrence and secondary cytoreductive surgery

	Endometrial Carcinoma(N%)	Uterine Sarcoma(N%)	P-value
Clinical symptoms at recurrence			0.826
None	26 (55.3)	19 (51.4)	
Symptomatic	21 (44.7)	18 (48.6)	
Ascites			0.725
No	41 (87.2)	34 (91.9)	
Yes	6 (12.8)	3 (8.1)	
Size of largest tumor (cm)			**0.001**
≤ 6	41 (87.2)	19 (51.4)	
> 6	6 (12.8)	18 (48.6)	
Number of recurrent tumors			0.186
One	24 (51.1)	13 (35.1)	
Several	23 (48.9)	24 (64.9)	
Sites of recurrence			
Central pelvis	20 (42.6)	27 (73)	
Lateral pelvis	4 (8.5)	11 (29.7)	
Pelvic lymphnodes	5 (10.6)	1 (2.7)	
Peritoneal dissemination	10 (21.3)	17 (45.9)	
Paraaortic lymphnodes	7 (14.9)	2 (5.4)	
Colon	3 (6.4)	7 (18.9)	
Small intestine	1 (2.1)	1 (2.7)	
Liver	1 (2.1)	0 (0)	
Adrenal gland	1 (2.1)	0 (0)	
Lung	3 (6.4)	2 (5.4)	
Chest wall mass	0 (0)	1 (2.7)	
Mediastinal lymph nodes	1 (2.1)	0 (0)	
Vaginal wall	8 (17)	1 (2.7)	
Vulva	2 (4.3)	0 (0)	
Inguinal lymphnodes	1 (2.1)	0 (0)	
Parietooccipital region	1 (2.1)	0 (0)	
Operative time, h (mean)	2.85 ± 1.564	3.44 ± 1.308	0.072
Intraoperative bleeding, ml (mean)	237.23 ± 265.254	478.92 ± 594.978	**0.015**
Haemoglobin drop, g/dl (mean)	1.39 ± 1.213	2.12 ± 1.711	**0.025**
Surgical procedures			
Exploratomy laparotomy with biopsy	2 (4.3)	0 (0)	
Pelvic lymphadenectomy	7 (14.9)	4 (10.8)	
Colectomy	7 (14.9)	2 (5.4)	
Rectectomy	4 (8.6)	4 (10.8)	
Ileostomy	1 (2.1)	1 (2.7)	
Bladder resection	0 (0)	3 (8.1)	
Ureteral stents placement	3 (6.4)	7 (18.9)	
Ureterostomy	0 (0)	1 (2.7)	
Ureterovesical reimplantation	2 (4.3)	0 (0)	
Partial ureterectomy	1 (2.1)	0 (0)	
Reconstruction of bladder with ileum	0 (0)	1 (2.7)	
Pelvic mass resection	6 (12.8)	16 (43.2)	
Oophorectomy or oophorosalpingectomy	2 (4.3)	8 (21.6)	
Paraaortic lymphadenectomy	10 (21.3)	4 (10.8)	
Repair of vena cava injury	1 (2.1)	0 (0)	
Omentectomy or Peritoneal dissemination resection	10 (21.3)	17 (45.9)	
Small-bowel resection	3 (6.4)	3 (8.1)	
Partial hepatectomy	1 (2.1)	0 (0)	
Adrenal tumor resection	1 (2.1)	0 (0)	
Appendectomy	2 (4.3)	1 (2.7)	
Vaginectomy or Vaginal stump resection	16 (34.0)	4 (10.8)	
Diaphragmatic tumor resection	1 (2.1)	0 (0)	
Partial urethrectomy	3 (6.4)	1 (2.7)	
Subcutaneous mass excision	1 (2.1)	0 (0)	
Vulvectomy	3 (6.4)	0 (0)	
Inguinal lymph node resection	1 (2.1)	0 (0)	
Abdominal mass resection	1 (2.1)	2 (5.4)	
Chest wall tumor resection	0 (0)	1 (2.7)	
Pulmonary lobectomy	2 (4.3)	0 (0)	
Mediastinal lymphadenectomy	1 (2.1)	0 (0)	
Parietooccipital tumor resection	1 (2.1)	0 (0)	
Complications			
None	35 (74.5)	28 (75.7)	
Bleeding	0 (0)	1 (2.7)	
Infection	3 (6.4)	4 (10.8)	
Intestinal fistula	0 (0)	1 (2.7)	
Urinary fistula	1 (2.1)	2 (5.4)	
Ileus	3 (6.4)	2 (5.4)	
Hydrothorax	1 (2.1)	0 (0)	
Lymphocyst	1 (2.1)	0 (0)	
Pelvic effusion	0 (0)	1 (2.7)	
Poor wound healing or herniation	6 (12.8)	1 (2.7)	
Thrombosis	1 (2.1)	0 (0)	
Hydronephrosis	1 (2.1)	0 (0)	
Peripheral neuropathy	0 (0)	1 (2.7)	
Complications that require surgery			
No	44 (93.6)	34 (91.9)	1.000
Yes	3 (6.4)	3 (8.1)	
Length of hospital stays, day (mean)	19.98 ± 6.883	24.84 ± 7.437	**0.016**
Residual disease			**0.010**
None	38 (80.9)	20 (54.1)	
> 0	9 (19.1)	17 (45.9)	
Neoadjuvent chemotherapy			0.252
No	44 (93.6)	37 (100)	
Yes	3 (6.4)	0 (0)	
Adjuvent therapy after SCS			
None	6 (12.8)	10 (27)	
Chemotherapy only	20 (42.6)	20 (54.1)	
Radiotherapy only	9 (19.1)	2 (5.4)	
Combined chemoradiotherapy	0 (0)	2 (5.4)	
Hormonal therapy	12 (25.5)	2 (5.4)	
Other	0 (0)	1 (2.7)	
Recurrence			**0.001**
No	21 (44.7)	4 (10.8)	
Yes	26 (55.3)	33 (89.2)	
Death			**0.007**
No	26 (55.3)	9 (24.3)	
Yes	21 (44.7)	28 (75.7)	

SCS, Secondary cytoreductive surgery

### Survival

The tumor outcome of cases with recurrent EC was better compared to those with recurrent US. The 2-year PFS and 5-year OS of cases with recurrent EC were 48.9% and 59.6%, respectively, compared with 27% and 33.3% of those with recurrent US (P = 0.002 and 0.006, respectively). In the recurrent EC group, the median OS was not reached, and the median PFS was 23 months. In the recurrent US group, the median OS was 15 months, and the median PFS was 7 months.

### Predictors of survival

#### Univariate and multivariate Cox regression analyses of survival after SCS in cases with EC (Table 3)

**Table 3:** Univariate and multivariate analyses of survival after SCS in patients with endometrial carcinoma

	Progression-free survival				Overall survival			
	Univariate		Multivariate		Univariate		Multivariate	
	HR (95%CI)	P value	HR (95%CI)	P value	HR (95%CI)	P value	HR (95%CI)	P value
Age at recurrence (years)		0.701				0.165		
< 60	1				1			
≥ 60	1.171 (0.522–2.630)				1.847 (0.777–4.392)			
ECOG performance status before SCS		0.719				0.385		
0	1				1			
1–2	0.802 (0.241–2.672)				1.620 (0.545–4.818)			
FIGO stage at initial diagnosis		0.629				0.396		
I/II	1				1			
III/IV	1.238 (0.520–2.948)				1.482 (0.597–3.675)			
Histology								
Endometrioid	1				1			
Serous	1.412 (0.329–6.065)	0.642			1.722 (0.395–7.510)	0.469		
Clear cell	5.879 (0.726–47.617)	0.097			7.544 (0.897–63.485)	0.063		
Carcinosarcoma	1.366 (0.405–4.609)	0.615			0.973 (0.224–4.235)	0.971		
Tumor grade		0.081				**0.014**		**0.002**
G1/G1-G2/G2	1				1		1	
G2-G3/G3	2.231 (0.907–5.488)				4.166 (1.339–12.962)		11.236 (2.446–51.623)	
Previous radiotherapy		0.896				0.750		
No	1				1			
Yes	0.931 (0.321–2.705)				1.194 (0.401–3.552)			
Clinical symptoms at recurrence		0.799				0.832		
No	1				1			
Symptomatic	0.904 (0.415–1.969)				0.911 (0.383–2.163)			
Size of largest tumor (cm)		0.058				**0.007**		**0.033**
≤ 6	1				1		1	
> 6	2.591 (0.968–6.94)				4.115 (1.472–11.499)		4.408 (1.128–17.229)	
Number of recurrent tumors		**0.017**		0.133		**0.024**		**0.004**
One	1		1		1		1	
Several	2.684 (1.189–6.057)		2.315 (0.744–6.927)		2.859 (1.145–7.138)		9.672 (2.032–46.025)	
DFI before SCS (months)		**0.026**		0.451		0.085		
< 12	1		1		1			
≥ 12	0.409 (0.186–0.898)		0.710 (0.291–1.732)		0.466 (0.196–1.110)			
Retroperitoneal lymph node metastasis		0.425				0.259		
No	1				1			
Yes	0.671 (0.253–1.786)				0.495 (0.146–1.681)			
Distant metastasis		**0.018**		0.399		**0.039**		0.183
No	1		1		1		1	
Yes	2.763 (1.188–6.426)		1.672 (0.506–5.524)		2.616 (1.052–6.508)		3.185 (0.579–17.537)	
Lung metastasis alone		0.396				0.493		
No	1				1			
Yes	2.402 (0.318–18.132)				2.023 (0.270-15.174)			
Peritoneal dissemination		**0.034**		0.564		**0.005**		0.381
No	1		1		1		1	
Yes	2.488 (1.071–5.781)		0.610 (0.114–3.271)		3.696 (1.498–9.120)		0.393 (0.049–3.169)	
Vaginal wall metastasis alone		0.133				0.193		
No	1				1			
Yes	0.036 (0.000-2.765)				0.037 (0.000-5.315)			
Ascites		**0.001**		0.302		**0.000**		0.522
None	1		1		1		1	
Yes	4.962 (1.884–13.069)		2.55 (0.431–15.094)		6.119 (2.229–16.794)		1.723 (0.325–9.128)	
Residual disease		**0.017**		0.263		**0.022**		0.401
None	1		1		1		1	
> 0	2.956 (1.216–7.187)		1.857 (0.628–5.495)		2.918 (1.170–7.280)		1.605 (0.532–4.849)	
Neoadjuvant chemotherapy before SCS		0.788				0.541		
No	1				1			
Yes	1.219 (0.288–5.168)				1.576 (0.366–6.794)			
Adjuvant therapy after SCS								
None	1		1		1		1	
Chemotherapy only	0.656 (0.238–1.810)	0.416	0.489 (0.148–1.613)	0.240	0.560 (0.198–1.583)	0.274	0.083 (0.017–0.416)	**0.002**
Radiotherapy only	0.054 (0.006–0.469)	**0.008**	0.105 (0.010–1.150)	0.065	0.069 (0.008–0.597)	**0.015**	0.053 (0.003–0.934)	**0.045**
Hormonal therapy	0.163 (0.043–0.623)	**0.008**	0.187 (0.029–1.196)	0.077	0.103 (0.020–0.538)	**0.007**	0.063 (0.006–0.648)	**0.020**

SCS, Secondary cytoreductive surgery; ECOG, Eastern Cooperative Oncology Group; FIGO, International Federation of Gynecology and Obstetrics; HR, hazard ratio; CI, confidence intervals; DFI, Disease-free interval

Univariate Cox regression analysis suggested that 6 factors were significantly associated with PFS, including the number of recurrent tumors (several) (HR = 2.684, P = 0.017), DFI before SCS (≥ 12 months) (HR = 0.409, P = 0.026), distant metastasis (HR = 2.763, P = 0.018), peritoneal dissemination (HR = 2.488, P = 0.034), ascites (HR = 4.962, P = 0.001), residual disease (> 0) (HR = 2.956, P = 0.017), and adjuvant therapy after SCS (radiotherapy only, P = 0.008; hormonal therapy, P = 0.008).

All variables with P < 0.05 in the univariate analysis were analyzed by the multivariate analysis. However, no significant factor was found.

In the univariate Cox regression analysis of the OS, tumor grade (G2-G3/G3) (HR = 4.166, P = 0.014), size of the largest tumor (> 6 cm) (HR = 4.115, P = 0.007), distant metastasis (HR = 2.616, P = 0.039), the number of recurrent tumors (several) (HR = 2.859, P = 0.024), peritoneal dissemination (HR = 3.696, P = 0.005), ascites (HR = 6.119, P = 0.000), residual disease (> 0) (HR = 2.918, P = 0.022), and adjuvant therapy after SCS (radiotherapy only, P = 0.015; hormonal therapy, P = 0.007) were significant factors.

And in the multivariate Cox regression analysis of the OS, tumor grade, size of the largest tumor, the number of recurrent tumors, and adjuvant therapy after SCS were independent prognostic factors.

#### Univariate and multivariate Cox regression analyses of survival after SCS in cases with US (Table 4)

**Table 4:** Univariate and multivariate analyses of survival after SCS in patients with uterine sarcoma

	Progression-free survival				Overall survival			
	Univariate		Multivariate		Univariate		Multivariate	
	HR (95%CI)	P value	HR (95%CI)	P value	HR (95%CI)	P value	HR (95%CI)	P value
Age at recurrence (years)		**0.004**		0.345		**0.006**		**0.006**
< 60	1		1		1		1	
≥ 60	4.265 (1.572–11.574)		2.153 (0.438–10.577)		3.511 (1.424–8.656)		3.868 (1.469–10.185)	
ECOG performance status before SCR		**0.031**		0.237		0.069		
0	1		1		1			
1–2	3.232 (1.113–9.388)		3.000 (0.485–18.550)		2.521 (0.932–6.821)			
FIGO stage at initial diagnosis		0.362				0.533		
I/II	1				1			
III/IV	1.575 (0.593–4.183)				1.408 (0.481–4.122)			
Histology								
Uterine leiomyosarcoma	1				1			
Low-grade ESS	0.566 (0.246–1.299)	0.179			0.610 (0.250–1.491)	0.279		
High-grade ESS	1.493 (0.430–5.185)	0.528			1.454 (0.415–5.095)	0.559		
Other	0.645 (0.187–2.222)	0.487			0.615 (0.140–2.693)	0.518		
Clinical symptoms at recurrence		0.776				0.814		
No	1				1			
Symptomatic	0.905 (0.456–1.797)				0.915 (0.435–1.924)			
Size of largest tumor (cm)		0.774				0.643		
≤ 6	1				1			
> 6	0.904 (0.455–1.798)				0.839 (0.398–1.767)			
Number of recurrent tumors		**0.016**		0.532		**0.036**		0.925
One	1		1		1		1	
Several	2.523 (1.187–5.362)		1.333 (0.541–3.287)		2.471 (1.063–5.742)		0.947 (0.308–2.910)	
DFI before SCS (months)		0.090				**0.034**		**0.024**
< 12	1				1		1	
≥ 12	0.540 (0.265–1.101)				0.414 (0.183–0.934)		0.341 (0.134–0.870)	
Retroperitoneal lymph node metastasis		0.820				0.616		
No	1				1			
Yes	1.182 (0.280–5.001)				0.599 (0.081–4.429)			
Distant metastasis		0.601				0.842		
No	1				1			
Yes	0.728 (0.221–2.394)				1.131 (0.339–3.775)			
Peritoneal dissemination		**0.004**		**0.007**		**0.021**		**0.006**
No	1		1		1		1	
Yes	2.926 (1.404–6.096)		3.118 (1.356–7.169)		2.458 (1.144–5.282)		3.672 (1.441–9.361)	
Ascites		0.498				0.534		
None	1				1			
Yes	0.662 (0.201–2.184)				0.633 (0.150–2.675)			
Residual disease		**0.002**		**0.026**		**0.017**		0.789
None	1		1		1		1	
> 0	3.239 (1.563–6.711)		2.662 (1.122–6.319)		2.531 (1.182–5.421)		1.138 (0.443–2.924)	
Adjuvant therapy after SCS								
None	1				1			
Chemotherapy only	0.753 (0.347–1.634)	0.472			0.652 (0.284–1.495)	0.312		
Radiotherapy only	0.832 (0.180–3.849)	0.814			0.444 (0.056–3.512)	0.441		
CCRT	0.338 (0.043–2.677)	0.304			0.562 (0.071–4.458)	0.586		
Hormonal therapy	0.312 (0.040–2.467)	0.270			0.402 (0.051–3.191)	0.389		
Other	14.008 (1.211-162.075)	0.035			2.497 (0.297–21.022)	0.400		

SCS, Secondary cytoreductive surgery; ECOG, Eastern Cooperative Oncology Group; FIGO, International Federation of Gynecology and Obstetrics; HR, hazard ratio; CI, confidence intervals; ESS, endometrial stromal sarcoma; DFI, Disease-free interval; CCRT, Combined chemoradiotherapy

In the univariate Cox regression analysis, age at recurrence (≥ 60 years old) (HR = 4.265, P = 0.004), ECOG performance status before SCR (1–2) (HR = 3.232, P = 0.031), the number of recurrent tumors (several) (HR = 2.523, P = 0.016), peritoneal dissemination (HR = 2.926, P = 0.004), and residual disease (> 0) (HR = 3.239, P = 0.002) were significantly associated with PFS.

According to the multivariate Cox regression analysis, peritoneal dissemination and residual disease after SCS significantly affected the PFS of recurrent cases with US.

In the univariate Cox regression analysis of the OS, age at recurrence (≥ 60 years old) (HR = 3.511, P = 0.006), DFI before SCS (≥ 12 months) (HR = 0.414, P = 0.034), the number of recurrent tumors (several) (HR = 2.471, P = 0.036), peritoneal dissemination (HR = 2.458, P = 0.021), and residual disease (> 0) (HR = 2.531, P = 0.017) were significant factors.

And in the multivariate Cox regression analysis of the OS, age at recurrence, DFI before SCS, and peritoneal dissemination were independent prognostic factors.

### Clinicopathological variables associated with complete cytoreduction

#### Univariate and multivariate logistic regression analyses of cases with EC (Table 5)

**Table 5:** Clinicopathological variables associated with optimal cytoreduction in patients with endometrial carcinoma

	Complete cytoreduction	Univariate		P value	Multivariate		P value
	(N%)	HR	95% CI		HR	95% CI	
Age at recurrence (years)							
< 60	26 (81.3)	1					
≥ 60	12 (80.0)	1.083	0.231–5.081	0.919			
ECOG performance status before SCS							
0	32 (78.0)						
1–2	6 (100.0)	NA	NA	0.579			
FIGO stage at initial diagnosis							
I/II	31 (88.6)	1			1		
III/IV	7 (58.3)	5.536	1.175–26.072	**0.030**	30.777	1.167-811.458	**0.040**
Histology							
Endometrioid	33 (86.8)	1			1		
Non-endometrioid	5 (55.6)	5.280	1.048–26.589	**0.044**	0.820	0.08–8.359	0.867
Tumor grade							
G1/G1-G2/G2	18 (94.7)	1					
G2-G3/G3	15 (78.9)	4.8	0.483–47.682	0.181			
Previous radiotherapy							
No	31 (79.5)	1					
Yes	7 (87.5)	0.554	0.059–5.173	0.604			
Clinical symptoms at recurrence							
No	21 (80.8)	1					
Symptomatic	17 (81.0)	0.988	0.229–4.264	0.987			
Size of largest tumor (cm)							
≤ 6	35 (85.4)	1					
> 6	3 (50.0)	5.833	0.946–35.988	0.057			
Number of recurrent tumors							
One	23 (95.8)	1			1		
Several	15 (65.2)	12.267	1.389-108.325	**0.024**	20.050	1.222-329.049	**0.036**
DFI before SCS (months)							
< 12	16 (76.2)	1					
≥ 12	22 (84.6)	0.582	0.135–2.515	0.468			
Retroperitoneal lymph node metastasis							
No	28 (75.7)						
Yes	10 (100.0)	NA	NA	0.172			
Distant metastasis							
No	30 (78.9)	1					
Yes	8 (88.9)	0.469	0.051–4.137	0.504			
Lung metastasis alone							
No	37 (80.4)						
Yes	1 (100.0)	NA	NA	1.000			
Peritoneal dissemination							
No	33 (89.2)	1			1		
Yes	5 (50.0)	8.250	1.638–41.546	**0.011**	1.833	0.049–69.231	0.744
Extended beyond the pelvis							
No	21 (95.5)	1			1		
Yes	17 (68.0)	9.882	1.123–86.985	**0.039**	0.386	0.013–11.546	0.583
Ascites							
None	36 (87.8)	1			1		
Yes	2 (33.3)	14.400	2.073-100.012	**0.007**	26.009	0.294-2301.929	0.154
Neoadjuvant chemotherapy before SCS							
No	35 (79.5)						
Yes	3 (100.0)	NA	NA	1.000			

SCS, Secondary cytoreductive surgery; ECOG, Eastern Cooperative Oncology Group; FIGO, International Federation of Gynecology and Obstetrics; HR, hazard ratio; CI, confidence intervals; DFI, Disease-free interval

Univariate analysis suggested that 6 factors were significantly associated with complete cytoreduction of SCS, including FIGO stage at initial diagnosis (III/IV) (HR = 5.536, P = 0.030), histology (non-endometrioid) (HR = 5.280, P = 0.044), the number of recurrent tumors (several) (HR = 12.267, P = 0.024), peritoneal dissemination (HR = 8.250, P = 0.011), extension beyond the pelvis (HR = 9.882, P = 0.039), and ascites (HR = 14.400, P = 0.007).

Multivariate analysis showed that FIGO stage upon initial diagnosis and the number of recurrent tumors were significant factors affecting the outcome of SCS.

### Univariate and multivariate logistic regression analyses of cases with US (Table 6)

**Table 6:** Clinicopathological variables associated with optimal cytoreduction in patients with uterine sarcoma

	Complete cytoreduction	Univariate		P value		Multivariate		P value
	(N%)	HR	95% CI			HR	95% CI	
Age at recurrence (years)								
< 60	17 (56.7)	1						
≥ 60	3 (42.9)	1.744	0.331–9.189	0.512				
ECOG performance status before SCS								
0	17 (53.1)	1						
1–2	3 (60.0)	0.756	0.111–5.149	0.775				
FIGO stage at initial diagnosis								
I/II	16 (50.0)	1						
III/IV	4 (80.0)	0.250	0.025–2.489	0.237				
Histology								
Uterine leiomyosarcoma	9 (47.4)	1						
Low-grade endometrial stromal sarcoma	6 (57.5)	0.750	0.169–3.327	0.705				
High-grade endometrial stromal sarcoma	2 (66.7)	0.450	0.035–5.843	0.542				
Other	3 (75.0)	0.300	0.026–3.427	0.333				
Clinical symptoms at recurrence								
No	10 (52.6)	1						
Symptomatic	10 (55.6)	0.889	0.244–3.243	0.858				
Size of largest tumor (cm)								
≤ 6	11 (57.9)	1						
> 6	9 (50.0)	1.375	0.376–5.032	0.630				
Number of recurrent tumors								
One	12 (92.3)	1				1		
Several	8 (33.3)	24.000	2.634-218.666		**0.005**	19.589	1.902-201.718	**0.012**
DFI before SCS (months)								
< 12	9 (40.9)	1						
≥ 12	11 (73.3)	0.252	0.061–1.047	0.058				
Retroperitoneal lymph node metastasis								
No	19 (54.3)	1						
Yes	1 (50.0)	1.187	0.069–20.539	0.906				
Distant metastasis								
No	18 (54.5)	1						
Yes	2 (50.0)	1.200	0.150–9.570	0.863				
Peritoneal dissemination								
No	15 (68.2)	1				1		
Yes	5 (33.3)	4.286	1.058–17.363	**0.041**		1.533	0.291–8.061	0.614
Extended beyond the pelvis								
No	9 (47.4)	1						
Yes	11 (61.1)	0.573	0.155–2.117	0.403				
Ascites								
None	18 (52.9)	1						
Yes	2 (66.7)	0.563	0.046–6.806	0.651				

SCS, Secondary cytoreductive surgery; ECOG, Eastern Cooperative Oncology Group; FIGO, International Federation of Gynecology and Obstetrics; HR, hazard ratio; CI, confidence intervals; DFI, Disease-free interval

Univariate analysis suggested that 2 factors were significantly associated with complete cytoreduction of SCS, including the number of recurrent tumors (several) (HR = 24.000, P = 0.005) and peritoneal dissemination (HR = 4.286, P = 0.041).

The results of multivariate logistic regression analysis showed that only the number of recurrent tumors was an independent factor affecting the outcome of SCS.

**Fig. 1:**
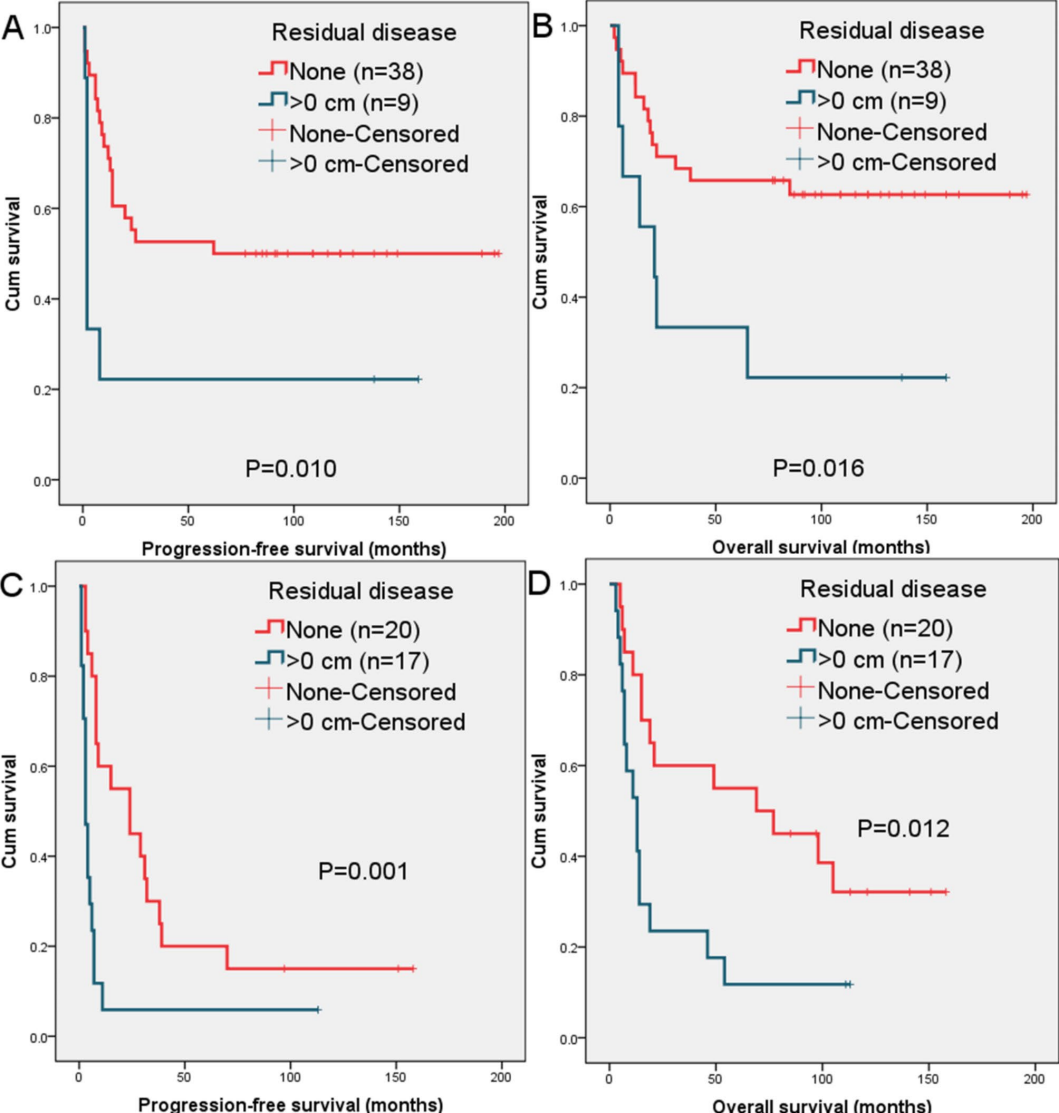
**A**. Comparison of progression-free survival curves in patients with recurrent endometrial carcinoma based on residual disease after secondary cytoreductive surgery; **B**. Comparison of overall survival curves in patients with endometrial carcinoma based on residual disease after secondary cytoreductive surgery; **C**. Comparison of overall survival curves in patients with recurrent uterine sarcoma based on residual disease after secondary cytoreductive surgery; **D**. Comparison of overall survival curves in patients with recurrent uterine sarcoma based on residual disease after secondary cytoreductive surgery

According to the Kaplan-Meier analysis, cases with recurrent EC and recurrent US who underwent SCS with no residual tumor had a longer survival (Fig. 1).

## Discussion

The mature of EC (including endometrioid carcinoma, serous carcinoma, clear cell carcinoma, mixed cell carcinoma, undifferentiated carcinoma, mesonephric adenocarcinoma, squamous cell carcinoma, mucinous carcinoma and carcinosarcoma) is different from US (including uterine leiomyosarcoma (uLMS), endometrial stromal sarcoma (ESS) and undifferentiated US (UUS), perivascular epithelioid cell tumor (PEComa), adenosarcoma (AS), rhabdomyosarcoma, etc.), so the two types were analyzed separately in our study.

In our study, endometrioid histology, FIGO stage I upon initial diagnosis, and good physical condition (ECOG score of 0) were more frequent in the cases with recurrent uterine malignancies who underwent SCS. Similarly, Moukarzel et al. [19] reviewed 376 cases with recurrent EC, the cases who underwent SCS had the longest survival (the longest OS: 57.6 months) and cases who were younger, or had stage I disease upon initial diagnosis, endometrioid histology, no residual disease after primary surgery, and longer interval to first recurrence or progression were more frequent among them. The results may state that patients with these characteristics can benefit from SCS.

We demonstrated that high histology grade was correlated with a shorter OS after SCS in cases with recurrent EC. Which was also showed in previous study, Ren et al. [6] showed that recurrent EC patients with high histology grade were related to a shorter OS after SCS. In addition, several studies stated histology was correlated with prognosis, which was not significant in our study. Shikama et al. [17] retrospectively reviewed 29 cases with recurrent EC who underwent SCS, the results of multivariate analysis showed that endometrioid histology and ECOG performance status score of 0 were significant and independent indicators of a longer OS. Similarly, in a study conducted by Odagiri et al. [20], histology was found associated with survival of cases with recurrent EC. The reason may be endometrioid histology accounts for the majority of patients in our study, the number of other histology type is too limited.

Compared with recurrent EC cases, multiple recurrent tumors, intraoperative blood loss, long-term hospitalization after SCS, large tumor size, and peritoneal dissemination were more common in cases with recurrent US. This may be related to the fact that the histopathological heterogeneity of US and uterine tumors was crushed in the abdominal cavity prior to the diagnosis of US. The surgical outcomes (complete cytoreduction) and tumor outcomes were poorer in cases with recurrent US.

For cases with recurrent US, peritoneal dissemination was found as an independent factor associated with shorter PFS and OS. Some studies also investigated recurrent ovarian cancer patients with mainly peritoneal dissemination were associated with poorer survival than those with lymph nodes metastasis. Just like it said above, partial peritoneal dissemination may be due to the primary surgery, it’s necessary to remove the specimen intactly or crush it in a protective bag before confirming the pathology of uterine tumor.

Moreover, our multivariate logistic regression analysis showed DFI before SCS (≥ 12 months) was correlated with a longer OS in cases with recurrent US. Similarly,Leitao et al. [12] and Bizzarri et al. [18] also demonstrated that a longer time to the first recurrence (> 12 months) was associated with an improved survival after SCS in cases with recurrent uLMS.

In our study, the univariate analysis showed that no residual disease after SCS was associated with longer PFS and OS for both cases with recurrent EC and US, while the multivariate analysis only showed that no residual disease was associated with longer PFS in cases with recurrent US, and this could be related to the limited sample size of our study. Several retrospective studies have also declared that the amount of residual tumor after SCS was an independent prognostic factor for survival of cases with recurrent EC and US. According to Papadia et al.’s findings [5], only absence of residual disease was associated with improved long-term outcomes of cases with recurrent EC. Shikama et al. [17] demonstrated that cases who underwent complete resection had a significantly longer OS after SCS than those who received incomplete resection. Awtrey et al. [4] also investigated cases with residual tumor ≤ 2 cm had a longer disease-specific survival after SCS. Leitao et al. [12] retrospectively analyzed data of 41 cases with recurrent uLMS who underwent SCS upon the first recurrence, and their results showed that optimal surgical resection was a predictor of improved outcomes. And a multi-institutional retrospective study suggested that SCS to no residual disease is an option that may be considered for cases with recurrent uLMS [18]. So, it is quite important to assess whether satisfactory tumor reduction can be achieved.

Single recurrent tumor was found as a significant and independent factor associated with complete cytoreduction both in cases with recurrent EC and US in our study. Previous studies investigated single recurrent tumor is the factor associated with optimal surgical resection but also showed other factors, like young age, tumor size < 6 cm, no peritoneal dissemination, and ECOG performance status score of 0 [6, 17].

Additionally, the rate of perioperative complications was approximately 25% in our study, and there were only 6 cases who required surgery (1 case of enterostomy, 1 case of puncture drain, 1 case of placement of vena cava filter, 1 case of cystostomy, and 2 cases of incisional hernia hernioplasty). According to the results of previous retrospective studies and findings of our study, the perioperative complications after SCS for cases with uterine malignancies were acceptable, and surgery can be considered for selected patients.

## Conclusion

Cases with recurrent EC had a better prognosis than those with recurrent US after SCS. In cases with recurrent EC, survival after surgery was longer for those with a lower tumor grade (G1/G1-G2/G2), size of the largest tumor ≤ 6 cm, single recurrent tumor, a history of adjuvant therapy. In cases with recurrent US, survival was improved for those with younger age, a longer DFI before SCS (≥ 12 months), no peritoneal dissemination. Complete cytoreduction is the goal of SCS, it may be performed in highly selected patients, with a single site of recurrence. However, larger sample size studies or prospective studies are needed to establish a model to determine which cases with recurrent uterine malignancies may benefit from SCS.

## Data Availability

The related data were available from the corresponding author upon reasonable request.

## Abbreviations

FIGO: International Federation of Gynecology and Obstetrics
ECOG: Eastern Cooperative Oncology Group
DFI: Disease-free interval
PFS: progression-free survival
OS: Overall survival
EC: Endometrial carcinoma
US: Uterine sarcoma
SCS: Secondary cytoreductive surgery
uLMS: Uterine leiomyosarcoma
ESS: Endometrial stromal sarcoma
UUS: Undifferentiated uterine sarcoma
PEComa: Perivascular epithelioid cell tumor
AS: Adenosarcoma
HR: Hazard ratio
CI: Confidence intervals
SPSS: Statistical Package for Social Sciences
